# Editorial: Bone marrow aging and its impact on immunosenescence in neurological diseases

**DOI:** 10.3389/fimmu.2025.1697136

**Published:** 2025-09-15

**Authors:** Junchi He, William G. Kerr, Xingzhi Guo, Dan Xu

**Affiliations:** ^1^ Department of Psychiatry, State University of New York Upstate Medical University, Syracuse, NY, United States; ^2^ Department of Biochemistry and Molecular Biology, State University of New York Upstate Medical University, Syracuse, NY, United States; ^3^ Department of Geriatric Neurology, Shaanxi Provincial People’s Hospital, Xi’an, Shaanxi, China; ^4^ Department of Emergency, Ruijin Hospital, Shanghai Jiao Tong University School of Medicine, Shanghai, China

**Keywords:** bone marrow aging, immunoscenecence, neurological disease, inflammaging, hematopoiesis

Aging is a fundamental biological process that profoundly affects multiple organ systems, among which the immune and nervous systems are particularly vulnerable (1). In neurological diseases such as multiple sclerosis, stroke, Alzheimer’s disease, and Parkinson’s disease, aging-related immune dysfunction (immunosenescence) has emerged as a critical factor influencing disease susceptibility, progression, and outcomes (2). Immunosenescence is characterized by chronic low-grade inflammation (“inflammaging”), impaired clearance of pathological proteins, and reduced capacity for neural repair (3). However, the origins and mechanistic pathways driving immunosenescence remain incompletely understood, limiting the development of targeted interventions.

Bone marrow aging has recently gained attention as a central contributor to immunosenescence (4). As the primary site of hematopoiesis, the bone marrow supports the production and functional maturation of immune cells that orchestrate both innate and adaptive immunity. Age-associated alterations in the bone marrow microenvironment, including hematopoietic stem cell dysfunction, changes in niche composition, and increased inflammatory signaling, disrupt immune cell homeostasis (5). These changes impair immune responses and subsequently affect the central nervous system’s ability to counteract neuroinflammation and promote repair. Despite its importance, the complex interplay between bone marrow aging, immunosenescence, and neurological disease pathophysiology has only begun to be elucidated.

This Research Topic gathers a collection of studies that explore these multifaceted relationships from molecular mechanisms to clinical observations, integrating insights from animal models, human case series, and comprehensive reviews. Collectively, these contributions advance our understanding of how bone marrow aging shapes immunosenescence and influences neurological health across the lifespan.

A mini review within this Research Topic provides a detailed examination of the cellular and molecular mechanisms linking bone marrow aging to immunosenescence and neuroinflammation (Müller and Di Benedetto). The authors highlight how age-related shifts in hematopoietic stem cell function and inflammatory signaling within the bone marrow microenvironment dysregulate immune cell production and function. This dysregulation fosters systemic immunosenescence and potentiates neuroinflammatory pathways implicated in age-related neurological disorders, including Alzheimer’s and Parkinson’s diseases. By synthesizing current knowledge, this review underscores the bone marrow’s role as a critical mediator in the pathophysiology of neurological diseases and highlights potential therapeutic targets aimed at preserving immune competence and neural integrity in aging populations.

Expanding the scope to neurodevelopmental disorders, an original research article investigates immune dysfunction and inflammation in aging Shank3b mutant mice, a model of autism spectrum disorder (ASD). The study demonstrates that aged Shank3b knockout mice exhibit elevated pro-inflammatory cytokines in the brain, bone marrow, spleen, and peripheral blood, accompanied by motor deficits (Cerilli et al.). These findings suggest that systemic “inflammaging” exacerbates ASD-related behavioral impairments, indicating immunosenescence is not confined to classical neurodegenerative diseases, but also influences neurodevelopmental conditions. This work broadens the impact of bone marrow aging and immune dysregulation, emphasizing the importance of considering immune aging across a diverse spectrum of neurological diseases.

Clinical insights are provided by a case series investigating thymic hyperplasia in multiple sclerosis (MS) patients after autologous hematopoietic stem cell transplantation (AHSCT) (Mariottini et al.). The study reveals radiological evidence of thymic reactivation in 20% of treated patients, suggesting that AHSCT promotes *de-novo* thymopoiesis and immune reconstitution. Given that premature thymic involution contributes to accelerated immunosenescence in MS, these findings underscore the thymus’s role as a pivotal organ to restore immune function. The reactivation of thymic activity post-AHSCT represents a promising mechanism to counter immune aging and improve clinical outcomes in autoimmune neurological diseases.

To complete this Research Topic, a comprehensive review explores neuroimmune dynamics throughout the lifespan, emphasizing the intricate bidirectional communication between the nervous and immune systems (Yeo et al.). Emerging evidence identifies a skull bone marrow reservoir supplying immune cells to the central nervous system, as well as anatomical interfaces such as the blood-brain barrier and meningeal system that facilitate neuroimmune crosstalk. This communication is shaped by both local tissue environments and systemic factors, enabling peripheral immune states to influence neurological function and vice versa. Yeo et al. discuss how aging impairs these feedback mechanisms, accelerating immunosenescence, neurological decline, and neuropathological progression. Understanding these intertwined changes offers critical insight for designing integrated therapeutic strategies that target both immune and neurological aging.

Together, these studies underscore the central role of bone marrow aging in driving immunosenescence and shaping neurological disease trajectories. They highlight the necessity of adopting a lifespan perspective that considers the dynamic interactions between hematopoiesis, immune function, and neurobiology. Future research harnessing advanced multi-omics technologies, sophisticated animal models, and longitudinal clinical studies will be essential to fully unravel these mechanisms. Such efforts hold promise for the development of precision medicine approaches to modulate immunosenescence and improve neurological health in aging populations.

We hope that this Research Topic will broaden readers’ perspectives and encourage them to explore the multi-faceted relationships linking bone marrow aging, immunosenescence, and neurological diseases.
